# Ultrasound diagnostics of renal artery stenosis: Stenosis criteria, CEUS and recurrent in-stent stenosis

**DOI:** 10.1007/s00772-015-0060-3

**Published:** 2015-08-28

**Authors:** W. Schäberle, L. Leyerer, W. Schierling, K. Pfister

**Affiliations:** Department of Visceral, Vascular, Thorax and Pediatric Surgery, “Klinik am Eichert”, Eichertstr. 3, 73035 Göppingen, Germany; Vascular and Endovascular Surgery, Regensburg University Hospital, Regensburg, Germany

**Keywords:** Color duplex ultrasonography, Renal artery stenosis, Stenosis criteria, Recurrent in stent stenosis, Contrast enhanced ultrasonography, Farbduplexsonographie, Nierenarterienstenose, Stenosekriterien, In-Stent-Rezidivstenose, CEUS

## Abstract

**Background and purpose:**

As a non-invasive, side effect-free and cost-effective method, ultrasonography represents the method of choice for the diagnosis of renal artery stenosis. Four different criteria in total, including two direct criteria in peak systolic velocity (PSV) and renal aortic ratio (RAR) and two indirect criteria in resistance index (RI) and acceleration time (AT) for the measurement of relevant renal artery stenosis are described, each demonstrating highly variable accuracy in studies. Furthermore, there is controversy over the degree beyond which stenosis becomes therapeutically relevant and which ultrasound PSV is diagnostically relevant in terms of stenosis grading.

**Material and methods:**

This article gives a critical review based on a selective literature search on measurement methodology and the validity of ultrasound in renal artery stenosis. A critical evaluation of methods and a presentation of measurement principles to establish the most precise measurement method possible compared with the gold standard angiography, as well as an evaluation of the importance of computed tomography angiography (CTA) and magnetic resonance angiography (MRA).

**Results and conclusions:**

The PSV provides high sensitivity and specificity as a direct measurement method in stenosis detection and grading. Most studies found sensitivities and specificities of 85–90 % for > 50 % stenosis at a PSV > 180–200 cm/s in ROC curve analysis. Other methods, such as the ratio of the PSV in the aorta to the PSV in the renal artery (RAR) or indirect criteria, such as side to side differences in RI (dRI) or AT can be additionally used to improve accuracy. Contrast-enhanced ultrasound improves accuracy by means of echo contrast enhancement. Although in the past only high-grade stenosis was considered relevant for treatment, a drop in pressure of > 20 mmHg in > 50 % stenosis (PSV 180 cm/s) is classified as relevant for increased renin secretion. Stenosis in fibromuscular dysplasia can be reliably graded according to the continuity equation. Although the available studies on the grading of in-stent restenosis are the subject of controversy, there is a tendency to assume higher cut-off values for PSV and RAR. Whilst MRA and CTA demonstrate an accuracy of > 90 %, this is at the cost of possible side effects for patients, particularly in the case of pre-existing renal parenchymal damage.

**Additional online material:**

This article includes two additional video sequences on visualizing renal artery stenosis. This supplemental material can be found under: dx.doi.org/10.1007/s00772-015-0060-3

## Zusammenfassung

**Hintergrund und Fragestellung:**

Als nichtinvasives, nebenwirkungsfreies und kostengünstiges Verfahren ist die Sonographie für die Diagnostik der Nierenarterienstenose die Methode der Wahl. Insgesamt vier verschiedene Methoden, zwei direkte („peak systolic velocity“, PSV, „renal aortic ratio“, RAR) und zwei indirekte Kriterien („resistance index“ RI, Akzelerationszeit) zur Messung relevanter Nierenarterienstenosen werden beschrieben, jeweils mit sehr unterschiedlichen Treffsicherheiten in verschiedenen Studien. Weiterhin wird kontrovers diskutiert, ab welchem Grad die Stenose therapierelevant ist, und welche sonographische Grenzgeschwindigkeit (PSV) in der Graduierung diagnostische Relevant besitzt.

**Materialien und Methode:**

Dieser kritische Review-Beitrag basiert auf einer selektiven Literaturrecherche zu Messmethodik und Validität der Sonographie bei Nierenarterienstenose. Es folgen eine kritische Methodenevaluation und Darstellung von Messprinzipien zur möglichst exakten Messung verglichen mit dem Goldstandard Angiographie sowie eine Bewertung des Stellenwerts der computertomographischen Angiographie (CTA) und Magnetresonanzangiographie (MRA).

**Ergebnisse und Schlussfolgerungen:**

Die PSV bietet als direkte Messmethode in der Stenosendetektion und Graduierung die höchste Sensitivität und Spezifität. Die meisten Studien ermitteln in ROC-Kurven bei einer PSV > 180–200 cm/s Sensitivitäten und Spezifitäten von 85–90 % für > 50%igen Stenosen. Andere Methoden wie der Quotient aus PSV in Aorta und Nierenarterie (RAR) oder indirekte Kriterien wie der Widerstandsindex im Seitenvergleich (dRI) oder die Akzelerationszeit können ergänzend zu Verbesserung der Treffsicherheit herangezogen werden. Die Kontrastmittelsonographie (CEUS) verbessert durch die Echokontrastverstärkung die Treffsicherheit. Früher wurden erst höhergradige Stenosen als therapierelevant angesehen, andererseits wird inzwischen schon der Druckabfall von > 20 mmHg bei > 50%igen Stenosen (PSV 180 cm/s) als relevant für einen Reninanstieg bewertet. Stenosen bei fibromuskulären Dysplasien können sonographisch nach dem Kontinuitätsgesetz zuverlässig graduiert werden. Die Studienlage für die Graduierung von In-Stent-Rezidivstenosen ist kontrovers, tendenziell höhere Cut-off-Werte für PSV und RAR sind jedoch anzunehmen. MRA und CTA zeigen zwar Treffsicherheiten von > 90 %, jedoch mit möglichen Nebenwirkungen für Patienten, insbesondere bei vorbestehendem Nierenparenchymschaden.

## Introduction

Renal artery stenosis (RAS) is found to be the cause of arterial hypertension in 1–5 % of patients [31] and is largely responsible for renal failure requiring dialysis in 5–15 % of patients [10, 13]. In addition to treatment for hypertension for which, however, there is no significant benefit compared with drug therapy, stent-assisted percutaneous transluminal angioplasty (PTA) is relevant in terms of organ and function preservation in high-grade RAS [18, 36].

Intra-arterial renal artery angiography is established as the gold standard for the diagnosis of RAS. A number of studies have evaluated the value of color-coded duplex ultrasonography (CCDS) for screening purposes [25, 50]. Magnetic resonance angiography (MRA) and computed tomography angiography (CTA) have also become established alongside CCDS. The latter is non-invasive, comparatively cost-effective, widely deployable and permits stenosis grading using hemodynamic measurement parameters; however, study results on stenosis grading are to some extent conflicting.

## Measurement methods

A total of four different methodological approaches to diagnosing RAS using CCDS have been evaluated over the last 25 years, 2 of which measure the degree of stenosis according to direct and 2 according to indirect criteria.

Direct criteria:

1. Peak systolic velocity (PSV) determines the degree of stenosis according to the continuity equation (PSV is inversely proportional to the cross-sectional area affected by stenosis and luminal reduction).
2. The ratio between PSV in the stenosed renal artery and PSV in the aorta (RAR renal aortic ratio) compares the increased intrastenotic flow velocity in the renal arteries with an individual reference value in the aorta. This approach attempts to reduce systemic influencing factors on PSV, such as current blood pressure; however, other factors having a hemodynamic effect on the aorta are difficult to evaluate.

Indirect criteria:

1. Poststenotic Doppler frequency spectra obtained from the renal hilum are evaluated. A reduction in the resistance index (RI) > 0.05 is an indication of ipsilateral RAS.
2. Delayed acceleration time (AT) distal to high-grade RAS, i.e. delay in systolic rise from end diastole up to PSV on spectral analysis.

As obtaining PSV in the proximal renal arteries can be challenging, some researchers prefer to do this at the renal hilum (indirect criteria). As is known from other vascular territories, indirect criteria only offer reliable accuracy in the case of high-grade stenosis. Although they demonstrate sufficient specificity in 50–70 % of cases depending on the method, sensitivity is poor at approximately ≤ 70 %. There is no consensus on the best method to detect RAS using CCDS as each method has its advantages and disadvantages; however, the significant variation in cut-off values above which both direct and indirect criteria assume a > 50 % or 60 % RAS is remarkable. Thus, peak flow velocities of 100–220 cm/s are given as the cut-off for 50 % stenosis using the most commonly used parameter, PSV. This achieves at times comparable, at times differing accuracies, a phenomenon that cannot be explained by study design alone.

## Examination procedure

**Fig. 1:**
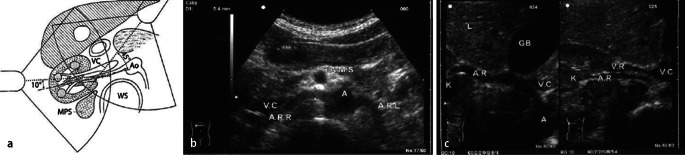
**a** Sketch of ultrasound visualization of the renal arteries, epigastric and flanking views [38] (*Ao* aorta, *GB* gall bladder, *k* kidney, *MPS* ileopsoas muscle, *WS* spine). **b** Sonoanatomy with visualization of both renal artery branches (*ARR* and *ARL*) originating from the aorta (*A*). Retrocaval (*VC*) course of the right renal artery (*ARR*) [38]. **c** Visualization of the entire course of the right renal artery (*AR*) from the renal hilum (*K*), along a retrocaval course (*VC*) to the aorta (*A*) in the *left image*; *right image*, the course of the right renal vein (*VR*) ventral to the artery (*AR*) [38]

When diagnosing stenosis using direct criteria (e.g. PSV and RAR), the examination takes place with the subject in a supine position. By dosing pressure with a transducer (2–5 MHz) it is possible to suppress artifacts from bowel gas and reduce the required penetration depth. The aorta is sought in cross-section from an epigastric approach and followed from the cranial to the peripheral aspect, where the renal arteries are located 1–3 cm distal to the mesenteric artery branch, which can be well localized in cross-section. Two structures that aid renal artery localization are the left renal vein, which courses between the aorta and the superior mesenteric artery to the vena cava. The right renal artery generally courses initially to the right in a ventrolateral direction (approximately 10–11 o’clock position) from the aorta, turns in an arc in a dorsal direction and then continues to run dorsal to the vena cava up to the right renal hilum (Fig. 1a, b). The left renal artery generally follows a lateral to dorsolateral course (approximately 3–5 o’clock position) from the aorta and then runs in its length of only 4–5 cm to the renal hilum.

Atherosclerotic stenosis is generally located at the origin of the renal artery. This can be the focus of the examination. Stenosis caused by fibromuscular dysplasia can be found in the middle third. To diagnose stenosis according to indirect criteria (e.g. AT and side to side differences in RI), the renal arteries are scanned on both sides in a flanking section and the renal arteries are visualized in the hilum. If patients are not overly adipose, the renal artery can also be probed in this way (transhepatic section) up to where it arises from the aorta in a right-sided paramedian section (banana peel view) (Fig. 1c). In order to obtain reliable values despite the relatively error prone measurement method when indirect criteria are used, the mean of multiple measurements (between three and five in total) needs to be taken.

## Significance of color-coded duplex sonography in the detection of renal artery stenosis

### Direct criteria

**Fig. 2:**
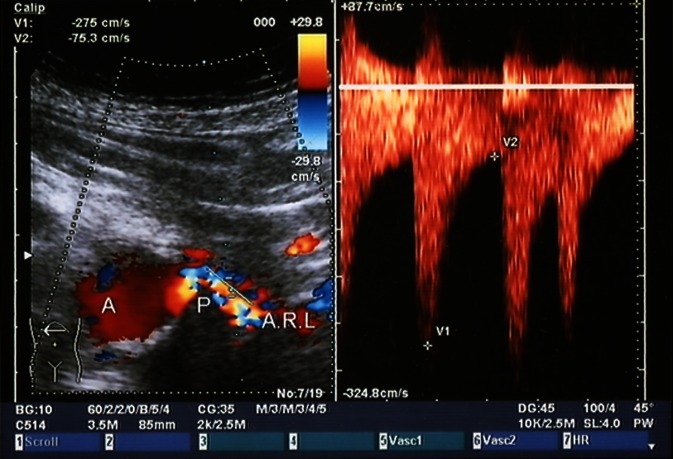
Left-sided renal artery stenosis (> 70 %) adjacent to the ostium (predilection site for atherosclerotic stenosis) with a PSV of 278 cm/s [38]

**Fig. 3:**
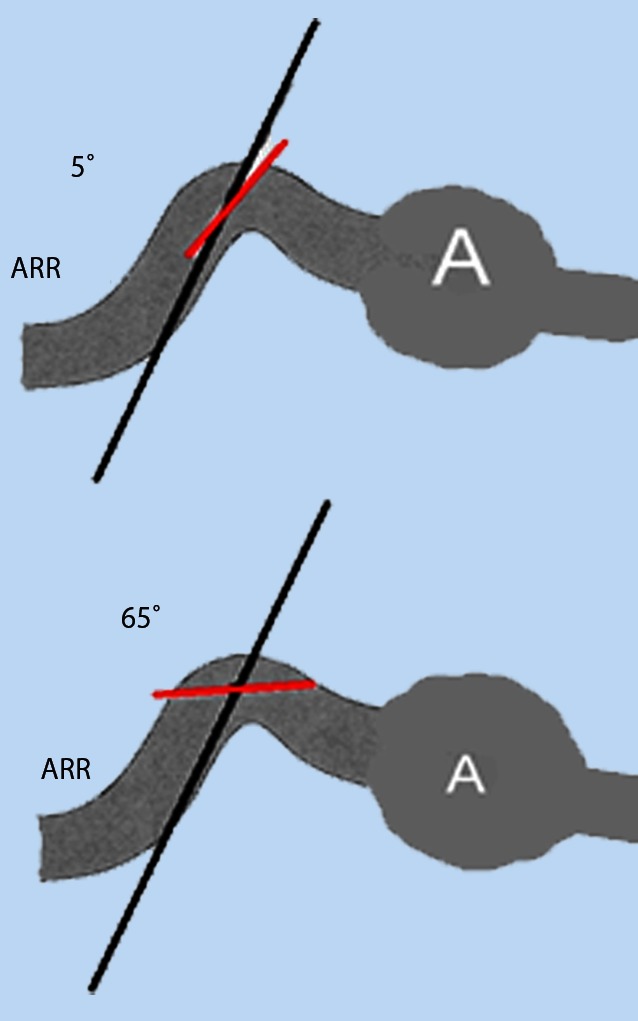
Difficulty associated with angle adjustment parallel to the flow vectors (vascular wall) in the case of a tortuous course of the renal artery at the origin of the artery (predilection site for atherosclerotic stenosis). At a Doppler angle of 65°, as little as ± 5° angle alignment errors can cause measurement deviations of over 30 % [38]

The significance of CCDS needs to be assessed in a differentiated manner relative to the criteria already described. Compared with the gold standard angiography, PSV demonstrates sensitivities of 71–98 % and specificities of 62–98 %, whereby some studies consider > 50 % and others > 60 % as hemodynamically relevant stenosis. The PSV cut-off ranges from 100 to 220 cm/s (Fig. 2). It should be noted here that older studies [2, 11, 16, 38] tended to set lower peak velocities (below 150 cm/s). These studies were not carried out using CCDS but using a combination of B-mode and Doppler sonography. The lack of color-coded vascular course (flow jet) has shown, also in our own experience, the risk of angle misalignment and hence measurement errors in the PSV approach (Fig. 3).

Studies conducted using CCDS (mostly after 1993) generally set the cut-off at 180–200 cm/s [8, 19, 24, 27, 40, 42]. As peak velocities are determined using receiver operating characteristics (ROC) curves, they also always represent an evaluation performed by the author to assess sensitivity or specificity, positive predictive value (PPV) or negative predictive value (NPV). Thus, a recent paper [1] reported a sensitivity, specificity, PPV, NPV and overall accuracy (OA) of 89 %, 54 %, 56 %, 88 % and 68 %, respectively, for a PSV of 200 cm/s. A PSV of 285 cm/s is set as the ideal cutoff for 60 % stenosis. Staub [42] described a PSV of 180 cm/s for 50 % stenosis with a sensitivity, specificity, PPV, NPV and OA of 96 %, 69 %, 81 %, 93 % and 85 %, respectively. A PSV of 200 cm/s yielded 92 %, 81 %, 87 %, 88 % and 87 %, respectively, while a PSV of 250 cm/s 78 %, 92 %, 93 %, 75 % and 84 %, respectively. This results in an ideal cut-off of 200 cm/s. Selecting higher PSV as cut-off values inevitably resulted in lower sensitivity and greater specificity in ROC curves compared with angiography and the converse in the case of a lower PSV. Other causes for differing PSV as peak velocities include the examination method, angle measurement errors (in particular due to the tortuous course of the proximal right renal artery) and the collective investigated (e.g. greater vessel wall rigidity and chronic renal parenchymal damage). Virtually none of the studies addressed systemic factors that influence PSV, such as current blood pressure and vessel wall rigidity. The PSV cut-off values are also strongly influenced by the little discussed problem of adequate stenosis grading in the reference method, angiography. The renal artery is difficult to visualize in the two planes necessary for appropriate grading (only oblique planes possible). Renal artery branch stenosis in particular can be challenging to visualize. Ultrasound PSV grading is generally compared with angiography solely in an anteroposterior plane. Although angiographic diagnosis offers good accuracy, there is poor concordance in stenosis grading between radiologists [49]. For this reason, an own study [38] used X-ray densitometry as an additional reference method and achieved a sensitivity of 86 % and a specificity of 83 % at a PSV cut-off of 140 cm/s. In addition, a good correlation (R = 0.84) between PSV and X-ray densitometry in stenosis grading of stenosed renal arteries before and after PTA was seen.

Particularly in the case of eccentric stenosis, significant discrepancies are observed between duplex ultrasonography and angiography, the latter showing a far lower hemodynamic effectiveness at the same angiographic diameter reduction compared with concentric stenosis (50 % diameter reduction in concentric stenosis = 75 % area reduction and 50 % diameter reduction in eccentric stenosis = 50 % area reduction). On duplex ultrasonography, the hemodynamic effectiveness of a stenosis is measured as an expression of area reduction. Thus, the PSV in concentric stenosis can be up to twice as high at the same diameter reduction (angiographic data) compared with eccentric stenosis [37].

An RAR of > 3.5 indicates an over 60 % RAS with a sensitivity of 84–91 % and a specificity of 95–97 % [16, 17, 22, 47]. Recent studies were unable to confirm this accuracy, working instead with sensitivities of 73–84 %, specificities of 72–81 % and accuracies of 76–78 % [1, 42]. End-diastolic peak velocity is sometimes also given as a stenosis criterion; however, as a parameter it depends heavily on heart rate and peripheral resistance and the findings, particularly in patients with early renal parenchymal damage cannot be used with sufficient accuracy as increased peripheral resistance causes reduced end-diastolic volume (EDV) early on.

**Fig. 4:**
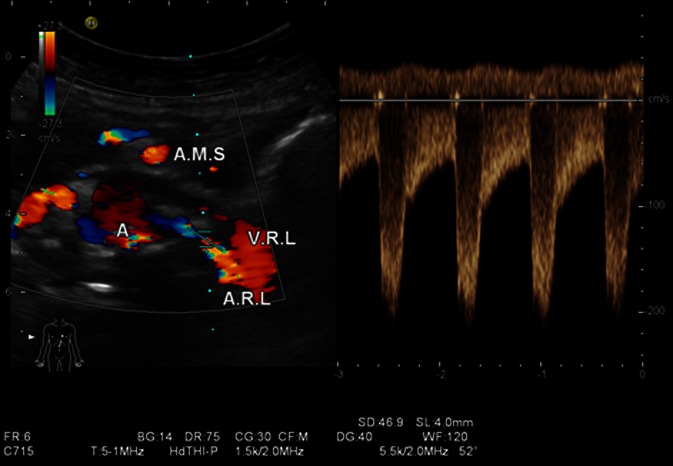
Fibromuscular dysplasia with 50–60 % stenosis in the middle third (predilection site). PSV ratio = 2.7 (see also video clip), PSV at the origin of the renal artery 80 cm/s and intrastenotic PSV 220 cm/s (stenosis grading according to the continuity equation)

**Fig. 5:**
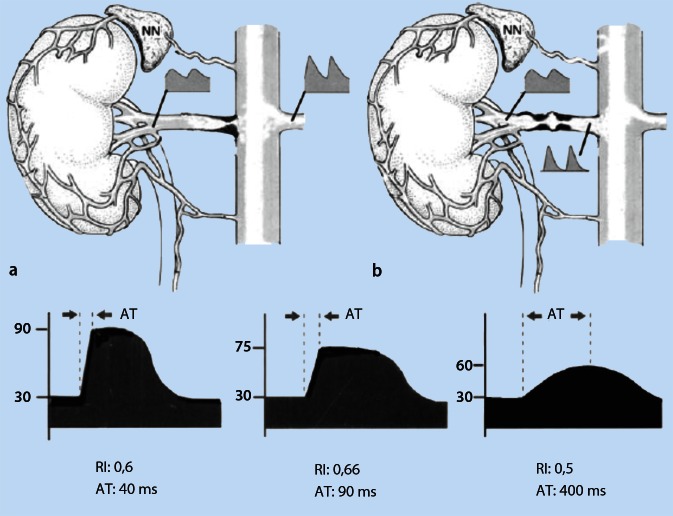
Poststenotic change in the Doppler frequency spectrum. **a** Comparison between poststenotic changes right and left. **b** Comparison of the Doppler frequency spectrum and RI before and after stenosis caused by fibromuscular dysplasia in the middle third (e.g. in the case of poor visualization of the middle third). **c** The increasing drop in the Pourcelot resistive index (RI = systolic PSV—end-diastolic PSV/systolic PSV) and the increase in acceleration time (*AT*) with increasing stenosis grade (*right* normal, *middle* moderate to high-grade stenosis, *right* > 90 % stenosis) [38]

There are currently no studies to validate CCDS specifically in stenosis caused by fibromuscular dysplasia. The main problem lies in the difficulty associated with visualizing the middle third on the left side as a result of gas artifacts from the colon. Comparing spectra and RI at the renal artery branch and at the hilum can be helpful here (see Fig. 5b). If visualization of the middle third is possible, stenosis grading can be reliably performed via the ratios (PSV ratio) between intrastenotic PSV and PSV in the first third (prestenotic) using the continuity equation (Fig. 4 and video clip 1). A PSV ratio of > 2 indicates a stenosis of > 50 % and > 4 of > 75 % (for concentric stenosis), offering greater grading reliability, as known from peripheral artery stenosis, compared with absolute PSV values.

### Indirect criteria

**Fig. 6:**
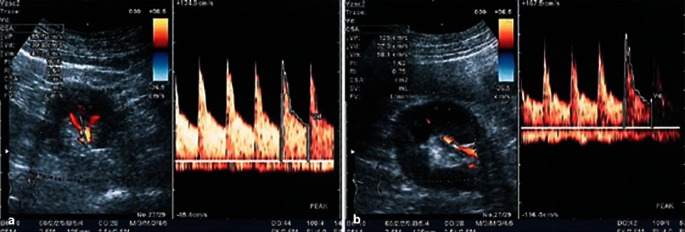
Comparison of the left-sided (RI = 0.64) and right-sided (RI = 0.75) resistance index for the detection of renal artery stenosis. Left RI > 10 % lower than right indicates left-sided stenosis (see Fig. 2)

Spectral analysis of vessels in other arterial territories reveals that indirect criteria only show measurement relevant lesions in the presence of high-grade stenosis. Thus, it is not surprising that a dRI of > 0.05 (Figs. 5 and 6) as the cut-off for 50 % stenosis has a sensitivity of 42 % and a specificity of 91 % (PPV 69 % and NPV 77 %). This poor sensitivity, even for > 70 % stenosis, has also been confirmed by Zeller [53] with a sensitivity of 77 % but a specificity of 99 %, as well as by Ripollés [33] with a sensitivity of only 50 % and a specificity of 90 % (PPV 69 % and NPV 92 %). Furthermore, Ripollés [33] made the interesting finding that a dRI > 0.05 could be used only in patients aged < 50 years with a sensitivity of 90 % and a specificity of 99 %. Poststenotic Doppler frequency spectra depend heavily on vessel rigidity and parenchyma function. The typical poststenotic changes (significantly reduced PSV in relation to EDV and prolonged AT) are not as marked in older patients with arteriosclerosis and renal parenchymal damage. On the other hand, a different resistance index (dRI) due to different degrees of renal parenchymal damage on the two sides results in measurement errors. The AT also confirms this with poor sensitivities (around 50 %) and good specificities (around 95 %) [8, 27] in < 80 % RAS.

## Treatment-oriented stenosis grading

Of the different stenosis criteria used a PSV > 180− 200 cm/s shows the best accuracy for the diagnosis and grading of RAS. To improve the inadequate sensitivities and specificities still seen in some studies, a number of authors recommend combining measurement results from a variety of (direct and indirect) stenosis criteria. In the study conducted by Staub et al. combining PSV and RAR yielded sensitivities and specificities of approximately 90 % [8, 24, 42] using a PSV of > 180 cm/s or 200 cm/s and an RAR of 3.5. Abu Rahma [1] described the combination of a PSV of > 285 cm/s and an RAR of > 3.5 as a viable criterion in renal artery diagnostics with a sensitivity of only 60 % but a specificity of 94 %, whereby this comparison was made with an angiographic stenosis of over 60 %. According to the ROC curve in the same study, a lower cut-off and the combination of a PSV of > 285 cm/s and an RAR of > 3.5 inevitably resulted in a significantly better sensitivity of 73 % but a worse specificity of 81 % (Table 1); however, the use of several stenosis criteria in the clinical routine is overly complex and time-consuming. For this reason, stenosis detection should be performed primarily by determining PSV and should only be complemented by RAR and dRI in doubtful situations or borderline findings.

**Table 1:** Color-coded duplex ultrasonography in the diagnosis of renal artery stenosis (RAS). Angiography-controlled studies on accuracy in hemodynamically relevant RAS. Various criteria are combined (direct and indirect stenosis criteria) to increase accuracy in RAS diagnosis

Author	Number (*N*) (stenosis degree)	Method/stenosis criterion	Sensitivity	Specificity	Reference method
Zeller et al. [53]	69 (> 70 %)	RAR > 3.5	100 %	60 %	Angiography
		dRI > 0.5	77.5 %	99 %	Angiography
		RAR > 3.5 and dRI > 0.05	76 %	97%	Angiography
Krumme et al. [24]	135 (> 50 %)	PSV > 200 and dRI > 0.05	89 %	92 %	Angiography
Motew et al. [27]	41 (> 60 %)	PSV > 180 cm/s	94 %	88 %	Angiography
		AT > 58ms	58 %	96 %	Angiography
		Conclusion: combination of methods recommended			
Ripollés et al. [33]	60 (> 75 %)	AT > 80 ms	89 %	99 %	Angiography
	Age < 50 years	AT > 80 ms	100 %	100 %	Angiography
	Age > 50 years	AT > 80 ms	75 %	97 %	Angiography
	Age < 50 years	dRI > 0.05	90 %	93 %	Angiography
	Ag > 50 years	dRI > 0.05	0 %	100 %	Angiography
		Conclusion: dRI and AT only useful as stenosis criterion in patients aged < 50 years			
Rademacher et al. [32]	226 (> 50 %)	PSV > 180 cm/s and PSV hilum < 1/4 PSV max. (Stenose) AT > 70 ms	96 %	98 %	Angiography
Souza de Oliveira et al. [41]	60 (> 50 %)	PSV > 150 cm/s	83.3 %	89.3 %	Angiography
Conkbayir et al. [8]	50 (> 60 %)	PSV > 180 cm/s	89 %	88 %	Angiography
		RAR > 3.0	86 %	97 %	Angiography
		AT > 70 ms	48 %	93 %	Angiography
		PSV > 180 cm/s or RAR > 3.0	92 %	88 %	Angiography
		PSV > 180 cm/s or RAR > 3.0 or AT > 70 ms	87 %	86 %	Angiography
		Conclusion: combination of methods recommended			
Kawarada et al. [20]	94 60%	PSV > 219	89 %	89 %	Angiography, transstenotic pressure gradient
Straub et al. [42]	49 (> 50 %)	PSV > 200	92 %	81 %	Angiographic stenosis degree, pressure gradient
		RAR > 3.0	83 %	91 %	Angiography
		dRI > 0.05	31 %	97 %	Angiography
	49 (> 70 %)	PSV > 250 cm/s	89 %	70 %	Angiography, angiographic stenosis degree, intra-arterial pressure measurement over stenosis
		RAR > 3.5	84 %	72 %	
		dRI > 0.05	42 %	91 %	
		PSV recommended possibly in combination with RAR (and dRI) to increase specificity			
Solar et al. [40]	94 60 %	PSV > 180	85 %	84 %	Angiography
Abu Rahma et al. [1]	313 60%	PSV180	91 %	41 %	Angiography
		PSV > 285	67 %	90 %	Angiography
		RAR > 3.5	72 %	81 %	Angiography
		PSV 180 + RAR 3.5	73 %	81 %	Angiography
		PSV 285 + RAR 3.5	60 %	94 %	Angiography
Schäberle et al. [40]	91 (> 50 %)	PSV > 140 cm/s	86 %	83 %	X-ray densitometry, angiography

*AT* acceleration time, *dRI* side to side differences in resistance index, *PSV* peak systolic velocity, *RAR* renal aortic ratio.

The use of CCDS with PSV, as well as the other criteria, shows greater accuracy for the detection of high-grade stenosis. It has hitherto been assumed that only stenosis > 70 % causes a relevant poststenotic pressure drop and increased renin release as a counterregulatory action via the renin-angiotensin system. Thus, only high-grade RAS are considered as requiring treatment [15, 26, 28, 48]. It is important to reliably identify these RAS for a treatment-oriented diagnosis. The discussion on the PSV cut-off at 50 % stenosis is more academic in nature than anything else; however, even at > 50 % RSA on angiography (Grosse et al. 2001) [42] measurements of the intra-arterial systolic pressure gradient show a mean pressure gradient of > 22 mm Hg. In Staub’s study [42] a PSV of > 200 cm/s (correlating with 50 % stenosis on angiography) showed a mean systolic pressure gradient of 23 mmHg and, hence, hemodynamically relevant stenosis accompanied by a regulatory counteraction [9]; however, it should be pointed out that poststenotic pressure was measured with the catheter lying across the stenosis (additional luminal narrowing).

The method for determining the pressure drop, as validated using the PSV in iliac stenosis [45] across the stenosis using the simplified Bernoulli equation (drop in pressure dP = 4 × intrastenotic PSV^2^) is only reliable in high-grade stenosis, as the prestenotic PSV is considered negligible in this context. In the case of renal artery branch stenosis, the prestenotic PSV in the aorta cannot be used. The poststenotic PSV [dP = 4 × (intrastenotic PSV^2–^ poststenotic PSV^2^)] occasionally used instead [46] of the prestenotic PSV (in the Bernoulli equation) is inaccurate and neglects inertial and frictional losses over the stenosis.

## Contrast-enhanced ultrasonography

A study including 120 patients with 38 stenosed renal arteries reported surprisingly good results [6]. Sensitivity, specificity, PPV, NPV and accuracy were reported as being 100 %, 84 %, 0 %, 80 % and 94 %, respectively, for CCDS compared with angiography in the same study. Claudon [7] described a 20 % improvement (from 63.9 % to 83.9 %) in stenosis detection in RAS using contrast-enhanced ultrasonography (CEUS) compared with conventional CCDS. In an already somewhat older study Missouris et al. found an increase in sensitivity from 85 % to 94 % and in specificity from 79 % to 88 % with a 20 dB increase in Doppler intensity following contrast medium administration.

## Ultrasound follow-up after stent placement

Using PSV and RAR in in-stent restenosis tend to show higher cut-off values at an equivalent degree of stenosis on angiography compared with native RAS [5, 12]; however, study results are conflicting. The explanation given for this in carotid artery restenosis is stent rigidity and a luminal reduction due to the stent. While Chi [5] set the ideal PSV cut-off for albeit > 70 % stenosis in stented renal arteries at > 395 cm/s and a RAR of > 5.1, Fleming [12] demonstrated a sensitivity, specificity, PPV and accuracy of 73 %, 80 %, 64 % and 77 % for > 60 % stenosis at a PSV of > 180 cm/s, 68 %, 80 %, 63 % and 76 % at > 200 cm/s and 59 %, 95 %, 87 % and 83 % at > 250 cm/s.

According also to these ROC curves, the selection of the ideal PSV cut-off should depend on the objective. If as much restenosis as possible is to be detected, a PSV of 180 cm/s should be selected due to its high sensitivity (73 %). If the focus lies on high-grade stenosis (where only this stenosis is considered relevant in terms of reintervention) a PSV with the highest PPV and specificity should be selected (87 % and 95 %, respectively for a PSV > 250 cm/s).

Controversially, Nolan [30] found similar stenosis velocity criteria for stented renal arteries compared with native stenosis (PSV > 200 cm/s and RAR > 3.5). Singh [34] also obtained the same results (PSV > 225 cm/s and RAR > 3.5 %). Napoli [29] even reduced the values for stented renal arteries (PSV from 180 cm/s to 144 cm/s and RAR from 3.5 % to 2.53 %) in order to improve sensitivity and specificity. Our own attempts to explain these results involved asking the question whether in-stent restenosis in this collective was more eccentric, which exhibits less area reduction and hence less hemodynamic relevance and lower PSV compared with concentric stenosis at equivalent angiographic diameter reduction.

**Fig. 7:**
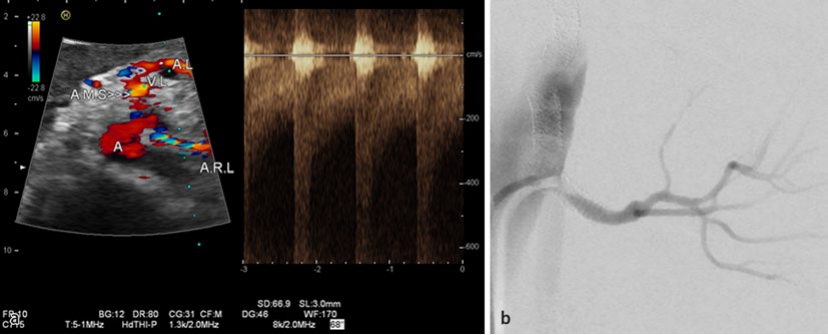
**a** High-grade left-sided in-stent restenosis (see also video clip) with a PSV of > 5.5 m/s and marked turbulence where the stent protrudes into the aortic lumen causing hyperechogenicity (*A* aorta, *AMS* superior mesenteric artery, *AL* splenic artery, *ARL* left renal artery, *VL* splenic vein). **b** Angiography of high-grade in-stent restenosis (probing at the origin of the renal artery stent protruding into the aortic lumen. Additional stent at the origin of the mesenteric artery projected on the aorta)

Besides the problems already described for native stenosis in stenosis detection using ultrasound compared with angiography, other weaknesses of poststenting studies lie in the low case numbers in generally retrospective, single center studies. Additional sources of error include a selection bias (angiographic follow-up only in clinically and sonographically proven pathological results), lack of information on the degree to which stenosis could be assessed (ultrasound conditions and angle alignment errors) and the impact of systemic factors on hemodynamics, meaning that some authors [5, 12] themselves questioned the possibility that their study results were a generalization (Fig. 7).

## Computed tomography angiography

The advent of multislice CTA and its ability to gather high-speed, thin-slice volume data sets has made it possible, in contrast to earlier technologies, to adequately assess the renal arteries. Although intra-arterial digital subtraction angiography (DSA) is still considered the gold standard, CTA overcomes the limitation whereby vessels (or luminal narrowing) can be visualized only intraluminally and provides images on wall calcification and lumen-narrowing plaque via three-dimensional data sets. Thus, studies conducted in recent years have shown sensitivities of 90–100 % and specificities of 92–98 % [4, 21, 35, 52]. Only the controversially discussed (study design) prospective multicenter Renal Artery Diagnostic Imaging Study in Hypertension (RADISH) has shown sobering results, with a sensitivity of 64 % and specificity of 92 %. Problems arise in the assessment of accessory renal arteries (pole arteries). With sensitivities of 100 % and specificities of 99 %, CTA yielded very good results in the assessment of in-stent stenosis following stent-assisted PTA in a study on 95 renal artery stents [44]. In addition to radiation exposure and possible contrast medium-induced nephropathy, particularly in patients with pre-existing parenchymal damage, the snapshot in time obtained with CTA and its inability to visualize hemodynamics, represents a further disadvantage.

## Magnetic resonance angiography

With sensitivities and specificities of 88–100 % [50], contrast-enhanced MRA is well suited as a non-invasive method to diagnose RAS; however, as known from other vascular territories, it overestimates the degree of stenosis by 26–32 % [14, 23, 43]. It also shows good accuracy in the visualization of accessory renal arteries [3, 14, 39, 51] as well as in the assessment of fibromuscular dysplasia [53]; however, the sensitivity was only 68 % for the grading of relevant stenosis in fibromuscular dysplasia.

In addition to a morphological assessment of the renal arteries, MRA also permits a functional assessment of the kidneys (renal parenchymal flow). Besides the known contraindications (i.e. nephrophathy and pacemakers), MRA is also susceptible to artifacts from neighboring metal or gas-containing organs, which can simulate stenosis. Furthermore, the broad application of gadolinium-enhanced MRA is limited due to the risk of nephrotoxicity and fibrosis, particularly in patients with impaired renal function and reduced glomerular filtration rate.

**Fig. 8:**
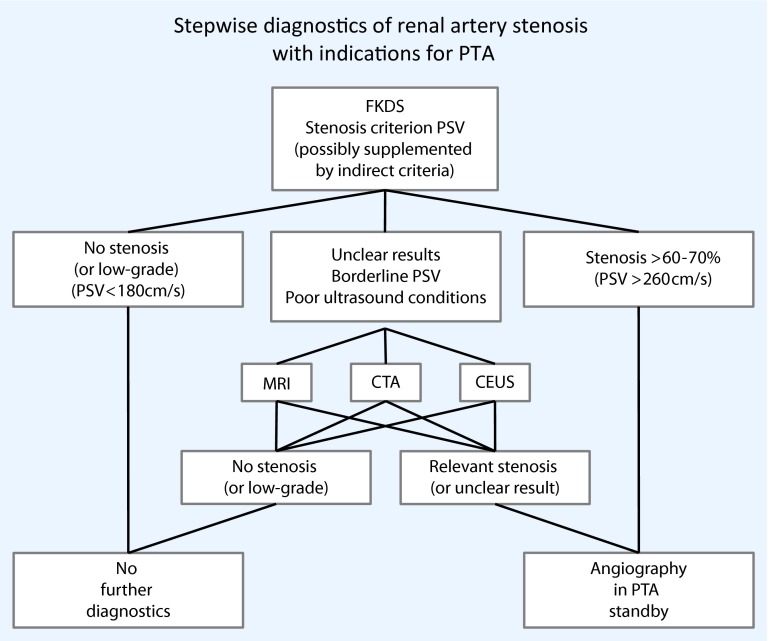
Stepwise diagnostics of renal artery stenosis (*CEUS*, contrast-enhanced ultrasonography, *CTA* computed tomography angiography, *FKDS color flow imaging* , *MRI* magnetic resonance imaging,*PSV* peak systolic velocity, *PTA* percutaneous transluminal angioplasty)

## Conclusion

Being a non-invasive, cost-effective and radiation-free examination method with good sensitivities and specificities (approximately 90 %), CCDS is well suited to the diagnosis of RAS. An intrastenotic PSV > 180− 200 cm/s represents the most accurate stenosis criterion for stenosis > 50 %. In the case of inconclusive findings, this criterion can be complemented by RAR or indirect criteria. It is possible to improve accuracies using CEUS. The MRA and CTA, with their good accuracy in the case of doubtful or borderline findings, offer an additional investigation method, albeit at the cost of higher patient exposure. Alternatively, when clinical findings are relevant to treatment, DSA can be performed on stand-by to convert to PTA (Fig. 8).

## Caption Electronic Supplementary Material

Video clip 1

(AVI 81026 kb)

Stenosis of 50–60 % in the middle third of the left renal artery due to fibromuscular dysplasia. Stenosis grading based on the continuity equation: PSV ratio where PSV intrastenotic/PSV prestenotic (at the origin of the renal artery) = 220 cm/s / 80 cm/s = 2.7. The video clip shows the intrastenotic PSV (in the middle third of the renal artery) and the prestenotic PSV (proximal third of the renal artery). Artery movement due to breathing complicates the measurement.

Video clip 2

(AVI 89551 kb)

High-grade renal artery recurrent in-stent stenosis after PTA and stent with PSV > 5 m/s. Images from conventional duplex ultrasound (B-mode image allows better visualization of the stent course) and color duplex ultrasound are both shown in an attempt to optimally display the stenosis jet for determination of the maximum PSV and therefore for adequate grading of the recurrent stenosis. Artery movement due to breathing complicates the measurement (the "sample volume" moves out of the vessel lumen/stent in a breathing-dependent manner). The stent (hyperechogenic, wire mesh pattern) protrudes slightly into the aortic lumen.
